# Randomized Waitlist-Control Trial of a Web-Based Stress-Management and Resiliency Program for Adolescent and Young Adult Cancer Survivors: Protocol for the Bounce Back Study

**DOI:** 10.2196/34033

**Published:** 2022-01-26

**Authors:** Helen Mizrach, Brett Goshe, Elyse R Park, Christopher Recklitis, Joseph A Greer, Yuchiao Chang, Natasha Frederick, Annah Abrams, Mary D Tower, Emily A Walsh, Mary Huang, Lisa Kenney, Alan Homans, Karen Miller, John Denninger, Ghazala Naheed Usmani, Jeffrey Peppercorn, Giselle K Perez

**Affiliations:** 1 Massachusetts General Hospital Boston, MA United States; 2 University of Massachusetts Memorial Medical Center Worcester, MA United States; 3 Dana-Farber Cancer Institute Boston, MA United States; 4 Connecticut Children’s Medical Center Hartford, CT United States; 5 University of Vermont Medical Center Burlington, VT United States

**Keywords:** cancer survivorship, adolescent and young adult (AYA), resiliency, stress management, coping

## Abstract

**Background:**

The emotional health of adolescent and young adult (AYA) cancer survivors is compromised both during and after cancer treatment. Targeted programs designed to support AYAs’ ability to cope with stress in the years following treatment completion are lacking. Mind-body programs may ameliorate the negative psychological and emotional effects of stress and assist AYAs with managing the psychosocial challenges of early survivorship.

**Objective:**

Our randomized waitlist-control trial aims to assess the feasibility, acceptability, and preliminary efficacy of a virtual group program (Bounce Back) to promote stress management and resiliency among posttreatment AYAs.

**Methods:**

Bounce Back is a stress management and resiliency program delivered via videoconference by a trained mental health clinician. Sessions were adapted from an evidence-based mind-body program (Stress Management and Resiliency Training - Relaxation Response Resiliency Program [SMART-3RP]) grounded in relaxation response elicitation, mindfulness, cognitive behavioral therapy, and positive psychology. Seventy-two AYAs (diagnosed with cancer between ages 14 years and 29 years and had completed cancer treatment within the last 5 years) were randomly assigned to the Bounce Back program or waitlist-control group and completed assessments at baseline, 3 months postbaseline, and 6 months postbaseline. The primary aim of the study is to determine the feasibility and acceptability of the Bounce Back program. Descriptive statistics, including means, frequencies, and ranges supplemented by qualitative exit interview feedback will be used to characterize the sample and to summarize feasibility and acceptability. The exploratory aims are to evaluate the preliminary effects of the program on stress coping and psychosocial outcome measures (ie, anxiety, depression) collected across the 3 time points.

**Results:**

This study was funded by the National Cancer Institute in July 2017. Study procedures were approved by the Dana-Farber Harvard Cancer Center Institutional Review Board in October 2018 (Protocol 18-428). The randomized trial was conducted from July 2019 to March 2021. Quantitative data collection is complete, and qualitative exit interview data collection is ongoing. Results are expected to be published in peer-reviewed journals and presented at local, national, or international meetings in the coming years.

**Conclusions:**

Few evidence-based programs exist that tackle the key transitional issues faced by AYA cancer survivors. Future analyses will help us determine the feasibility and acceptability of the Bounce Back program and its impact on AYA stress coping and psychological well-being.

**Trial Registration:**

ClinicalTrials.gov NCT03768336; https://clinicaltrials.gov/ct2/show/NCT03768336

**International Registered Report Identifier (IRRID):**

DERR1-10.2196/34033

## Introduction

Adolescence and young adulthood are life stages marked by peak physical, social, and emotional development. A cancer diagnosis and treatment during this stage can significantly disrupt many key life domains [1]. Adolescent and young adult (AYA) cancer survivors include individuals who are diagnosed with cancer between the ages of 15 years and 39 years. Approximately 89,000 AYAs are diagnosed with cancer annually, and cancer is the leading cause of disease-related deaths among individuals in this age range [2]. According to a recent systematic review, AYAs with cancer have reported difficulties with employment, educational attainment, and financial stability after treatment completion [3]. They also have challenges identifying their social support systems and report problems developing and maintaining peer, family, intimate, and marital relationships [3]. These challenges may impact their psychological well-being as they transition into the early survivorship period.

The emotional health of AYAs can be significantly compromised both during and after cancer treatment. Among AYAs with a history of cancer, stress has been linked to decreased physical activity and increased rates of drinking alcohol, smoking tobacco, and substance use [4,5]. Stress has also been shown to exacerbate the posttreatment symptoms AYAs frequently experience, including pain, fatigue, and insomnia [6]. Their health-related quality of life may be poor, and they experience elevated levels of distress posttreatment [7-10]. Although acute distress symptoms can persist for several years after treatment, peak levels of distress typically coincide with the first few years of treatment completion [10,11]. These consequences combined may increase AYAs’ risk for cancer-related morbidity and early mortality, yet targeted programs to support AYAs’ ability to cope with stress in the years following treatment completion are lacking [12-17].

Mind-body programs, which teach skills to improve the connection between the mind and body (ie, yoga, tai chi, mindfulness training), may ameliorate the negative psychological and emotional effects of stress and help AYAs manage the psychosocial challenges of early survivorship [18-21]. AYAs have shown interest in using complementary and alternative medicine, which encompasses mind-body approaches, to cope with stress and improve overall well-being [22-24]. However, there are few established programs demonstrating the utility of these approaches for AYAs during the early survivorship period [14,15,25,26].

Here, we describe the protocol for a pilot randomized waitlist-control trial of a scalable virtual group program (Bounce Back) aimed at promoting stress management and coping among posttreatment AYA cancer survivors. With funding from the National Cancer Institute (NCI), we adapted Bounce Back from an existing evidence-based resiliency program, the Stress Management and Relaxation Training - Relaxation Response Resiliency Program (SMART-3RP) [27]. Our program adaptation was informed by a series of qualitative focus groups with AYAs and open pilot testing for program refinement. Based on our qualitative data, we modified the program content to “normalize” the posttreatment challenges (eg, returning to school or work, socializing with peers again) common to the AYA experience. Bounce Back aimed to prevent the emergence of anxiety and depressive symptoms in AYAs by introducing stress coping skills early in the posttreatment experience [28]. To our knowledge, Bounce Back is the first stress management and resiliency program targeting the early posttreatment stressors of AYAs.

The Bounce Back study was a partnership between Massachusetts General Hospital (MGH), the Dana-Farber Cancer Institute (DFCI), and the Consortium for New England Childhood Cancer Survivors (CONNECCS [29]). CONNECCS consists of 14 pediatric cancer clinics located across 6 New England states (Connecticut, Maine, Massachusetts, New Hampshire, Rhode Island, and Vermont). The primary aim of the study is to determine the feasibility and acceptability of the Bounce Back program. The exploratory aims are to evaluate the preliminary effects of the program on stress coping and psychosocial outcome measures (ie, anxiety, depression, intolerance of uncertainty) collected across 3 time points.

## Methods

### Ethics Approval

Study procedures were approved by the Dana-Farber Harvard Cancer Center IRB in October 2018 (Protocol 18-428).

### Study Design

**Figure 1:**
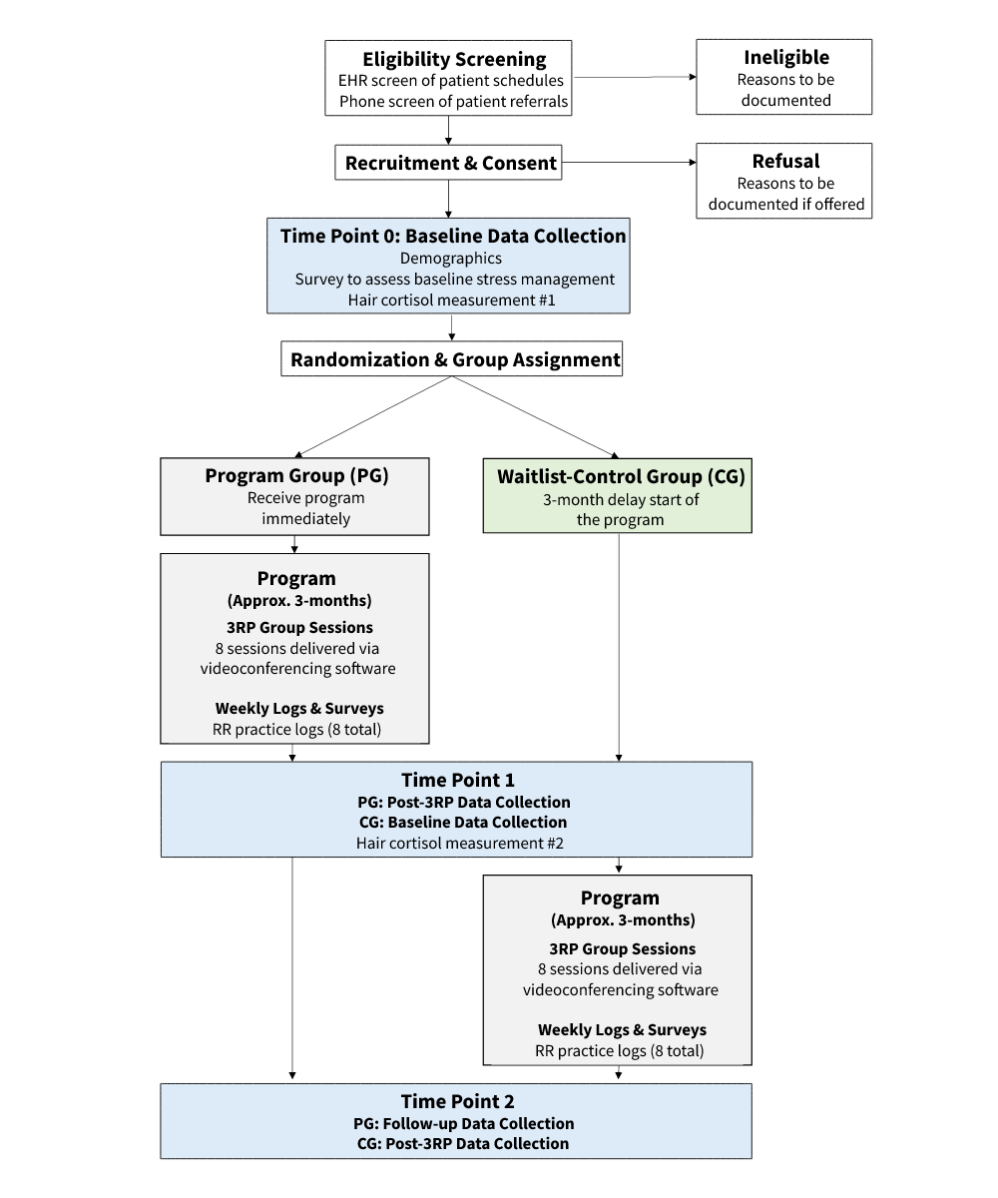
Participant flow. 3RP: Relaxation Response Resiliency Program; EHR: electronic health record.

The study was designed as a pilot randomized waitlist-control trial examining the feasibility, acceptability, and preliminary efficacy of Bounce Back delivered during early posttreatment for AYA cancer survivors. Eligible participants were randomly assigned to the Bounce Back program group (PG) or waitlist-control group (CG) and were asked to complete assessments at 3 time points: baseline, 3 months postbaseline, and 6 months postbaseline. Prior to study start, the Dana-Farber Harvard Cancer Center Institutional Review Board (IRB) reviewed and approved the study protocol and consent forms (Protocol 18-428). Recruitment occurred from May 2019 to September 2020. Figure 1 illustrates the overall design and participant flow of the study.

### Participants

Eligible participants included survivors of cancer diagnosed during early adolescence and young adulthood (ages 14-29 years) who had completed treatment within the past 5 years and who were between the ages of 16 years and 29 years at study enrollment (Table 1). We defined treatment completion as the date of the last intensive cancer treatment session (eg, chemotherapy, surgery, radiation) with curative intent. AYAs who were within 5 years of completing cancer treatment and did not have evidence of residual disease but who were receiving maintenance or hormonal treatment (eg, rituximab, tamoxifen) were considered eligible for the study. Given the virtual trial design, AYAs were eligible to participate if they spoke and read English and were able to connect to group sessions via the videoconferencing software. Of note, there were no entry criteria related to the presence of emotional distress.

**Table 1:** Study eligibility criteria.

Criteria		Rationale
**Inclusion criteria**		
	Diagnosed with any cancer between ages of 14 years and 29 years	To target AYAs^a^ diagnosed during a time of significant developmental change; age range also within the focal age range identified by the National Clinical Trials Network–affiliated Children Oncology Group scientific committees that focus on AYA cancer [30]
	Completed cancer treatment within the past 5 years	Opportunity to address stressors associated with early posttreatment survivorship; this window for treatment completion consistent with the “early survivorship” period, when concern about recurrence is high [31]
	Between 16 years and 29 years of age at time of enrollment	Optimize AYA heterogeneity in terms of life stage; also includes individuals likely to experience insurance changes
**Exclusion criteria**		
	Unable to speak or read English	Limited to English speakers due to breadth and exploratory nature of the study
	Is medically or otherwise unable to participate (as determined by a physician or study principal investigator)	For safety, due to virtual nature of the program
	Unwilling or unable to participate in study sessions delivered via the Zoom videoconferencing software	Program only offered via videoconference technology

^a^AYAs: adolescents and young adults.

### Recruitment

A multimodal approach was used to identify potential AYA participants for this study.

#### Provider Referral

Clinicians at MGH, DFCI, CONNECCS-affiliated sites, and external health care institutions could present and recommend the study to AYAs during a regular clinic visit. To facilitate provider support and referral, the study principal investigator (PI) presented the trial and solicited provider input at each of the Mass General Brigham and CONNECCS-affiliated sites. Interested patients either contacted the study clinical research coordinator (CRC) directly or provided permission to have the study CRC reach out to them.

#### Proactive Electronic Health Record Screening

The study CRC screened cancer survivors’ electronic health records (EHR) at MGH for demographic and clinical eligibility criteria. Following patient identification, the CRC requested permission from a member of the cancer care team (ie, oncologist, nurse practitioner) to approach the patient for study participation at their next scheduled clinic visit or pursue outreach via phone. DFCI study staff conducted a chart screen and transferred the names and contact information of potentially eligible participants to MGH using a secure study REDCap (Research Electronic Data Capture) database to allow for the CRC to assess eligibility and pursue outreach.

#### Recruitment Flyers

Recruitment flyers were distributed through social media; by providers at external, interested health care institutions and clinics, including the CONNECCS network; and at cancer and survivor-related conferences and organizations (eg, Stupid Cancer; Cancer Con).

#### Research Portals

The Bounce Back study appeared as a public page on the Mass General Brigham Rally Research Recruitment Portal, a tool that allows patients to express interest in ongoing research studies.

#### Social Media Recruitment

Social media advertisements were used to disseminate information about the Bounce Back study and to direct AYAs to contact the study team for more information. Both recruitment flyers and text posts were posted on a variety of social media outlets and forums including Reddit, Facebook, Instagram, and Twitter.

### Outreach and Follow-up Procedures

Patients who directly reached out to express interest in the study through recruitment flyers, research portals, or social media were contacted by the CRC within 1 to 2 business days of their initial outreach. Patients who were referred by providers or identified by proactive EHR screening were contacted by the CRC within 1 week after receiving permission from their health care provider(s). Phone calls, voicemails, and recruitment emails were also utilized as initial outreach methods.

For the initial phone outreach, the CRC followed an IRB-approved phone script to introduce the study and gauge interest in participating. Interested AYAs proceeded to complete the eligibility screening process.

During initial email outreach, the CRC sent an IRB-approved templated email to potential participants containing a brief overview of the study and a PDF of the study recruitment flyer. AYAs were prompted at the end of the email to reply if they were interested in enrolling or learning more information. They were also given the options to decline participation and decline to receive further communication. The CRC then assessed eligibility and completed the electronic informed consent process via phone or a Zoom call.

Often, referring providers shared contact information for an AYA with the study team, which turned out to be parental contact information instead of the AYA survivors’ personal contact information. On these occasions, outreach proceeded with contacting the parent. For parents of AYAs under the age of 18 years, the CRC encouraged co-participation of the AYA at the initial call. For parents of AYAs over the age of 18 years, the CRC asked the parent to provide the AYA’s phone number or email address so they could pursue direct outreach.

Up to 3 repeated contact attempts were made using the aforementioned outreach methods.

### Screening

To confirm eligibility prior to enrollment, the CRC administered a series of screening questions to all interested AYAs over the telephone or Zoom videoconferencing. During eligibility screening, all individuals were asked to verify (1) their date of birth, (2) their date of cancer diagnosis, and (3) details regarding their cancer treatment history (eg, date of treatment completion) and trajectory (eg, no further treatment planned apart from surveillance, no evidence of disease but use of rituximab).

### Consent

Once the CRC confirmed an AYA’s eligibility, they obtained informed consent using an electronic research consent form hosted on MGH REDCap. Participants were informed of the program components in greater detail, the required and optional assessments, potential risks and benefits of study participation, and the breakdown of the study compensation (up to US $120). They were also informed of the approximate start dates of the next 2 scheduled Bounce Back groups; groups were run consecutively so they would be later randomized to join one of the 2 next groups. Consented participants were emailed a PDF copy of the consent form for their records.

For participants under 18 years old, the CRC explained the study procedures to both the individual and their parent or legal guardian concurrently, and assent was obtained by the minor participant and their parent.

### Enrollment

After informed consent, participants were assigned a study ID number, which was linked to their baseline assessment survey. To standardize the date of survey completion between groups, the baseline survey was sent approximately 2 weeks before the start of a new group to all participants who had consented. Participants were considered “enrolled” in the study following completion of the baseline survey (T0) and after randomization.

### Randomization

Participants were randomized to the Bounce Back PG or CG following completion of the baseline survey (T0). Study staff developed a computer-generated randomization schema and stored condition assignments in concealed envelopes. Envelopes containing the randomization assignment were opened by the CRC while on the phone with participants.

### Participation Timeline

Participants randomized to the PG initiated the Bounce Back program in the next scheduled group. After program completion, they completed a posttreatment questionnaire (T1) to examine pre-post treatment changes in exploratory measures and a 3-month follow-up (T2) questionnaire to examine potential maintenance of program benefits (by evaluating change in scores from T1 to T2).

Participants randomized to the CG enrolled in the study and completed the baseline survey (T0) at the same time as the PG. They then completed the baseline a second time (T1) after the PG completed the Bounce Back program to allow for pre-post treatment group comparisons (T0 vs T1). After program completion, the CG completed a posttreatment assessment to examine pre-post treatment changes in exploratory measures (T1-T2).

### Participant Communication Methods

Previous literature has shown that recruiting AYAs for research studies can be difficult [32-34]. Informed by our previous work, we maintained contact with participants through communication methods with which they were comfortable and familiar, including phone, secure videoconferencing (eg, Zoom), email, and SMS texting [28].

### Preprogram

To facilitate proficiency, familiarity, and comfort with the Zoom videoconferencing software, participants were required to meet with the CRC for a 10-15–minute Zoom test call approximately 1 week prior to the start of the program. During these test calls, the CRC provided an overview of the Bounce Back program (ie, surveys and hair samples) and addressed any remaining questions or concerns. Participants were also offered the opportunity to have a brief individual meeting with the group facilitator prior to the first group session. These optional, 15-minute meetings were designed to establish rapport, review group expectations, and address any remaining concerns about participating in an online virtual group. The CRC documented the number of participants who completed test calls and optional pre-program group facilitator meetings along with reasons for refusal.

### Treatment Overview

The SMART-3RP program [27] was adapted to create the Bounce Back program, which was designed for virtual clinician-directed delivery over videoconference to groups of AYAs. Program adaptations were informed by reviews of the literature identifying gaps in posttreatment care for AYAs, meetings with AYA experts and clinicians, and focus groups and interviews with AYAs [28]. In Bounce Back, topics relevant to AYAs were interwoven throughout the program and used as a guide for applying techniques to relatable challenges. For instance, social and educational topics identified in qualitative interviews [28], such as how to tell friends about their cancer experience, having empathy for “small things,” relating to others postcancer treatment, preparing for high school or college, and managing parents’ anxieties, were interwoven throughout the program and used to guide survivors in applying learned skills (eg, identifying types of social support needed and developing strategies to facilitate social outreach and connection). Participants were emailed a PDF copy of the next chapter of the Bounce Back treatment manual the day before each weekly session to follow along with the program content. Please see Table 2 for a session-by-session overview of the Bounce Back program.

**Table 2:** Bounce Back program session-by-session content.

Program session	Educational content	Exercises and skills
Session 1: Stress Management and Resiliency Training	Group member introductions The science of mind-body medicine Components of Bounce Back (practicing relaxation response [RR] techniques, stress awareness, adaptive strategies) Breath awareness	Body awareness Photography as RR RR practice: simple breath awareness
Session 2: The RR	A closer look at the RR Appreciations Components of the stress response Sleepiness vs fatigue The MINI: an RR tool to use in the moment	RR practice: autogenic training Stress warning signals Fatigue warning signals RR practice: MINIs
Session 3: Stress Awareness	Mindful awareness Awareness of emotions and physical sensations Social support Changes in the self before and after cancer Mindful eating exercise	RR practice: mindful awareness Mindful eating Identifying emotions and positive physical sensations The social support diagram I am “Me”
Session 4: Mending Mind and Body	Awareness of movement Negative automatic thoughts Pleasant activities Values	RR practice: yoga Coping log Reflecting on what’s important MINI: walking meditation
Session 5: Creating an Adaptive Perspective	Guided imagery Coping strategies: acceptance versus problem solving Promoting physical activity	RR Practice: Insight Imagery Creating Adaptive Perspectives
Session 6: Promoting Positivity	Contemplation Optimism versus pessimism Healthy eating after cancer	RR practice: contemplation Comparing optimism and pessimism Relaxation signals
Session 7: Healing States of Mind	Empathy and compassion Self-empathy Creative expression	RR practice: compassion meditation Root fear Poetry
Session 8: Humor and Staying Resilient	Humor and coping Laughter Humor strategies Staying resilient: plan for long-term resiliency	RR practice: idealized self Energy battery 2 Finding humor in your life Laughter Empathy: relating to others

Throughout the program, participants were encouraged to practice RR strategies at home for at least 10 minutes to 20 minutes each day. To facilitate practice, participants received mailed copies of weekly relaxation response (RR) practice logs before the start of the program as well as weekly electronic practice logs following each session. The physical and electronic practice logs were identical. Both included questions about weekly RR elicitation and appreciations, as well as stress, distress, and coping Likert scales. AYAs were encouraged to document the frequency and duration of their RR practice each week on either the paper copy or electronic copy of the practice log, as per their preference.

### Treatment Administration

The Bounce Back program consisted of 8 weekly, 90-minute sessions delivered virtually by a clinician via Zoom videoconferencing software. Groups consisted of approximately 8 participants (mean 8, range 4-10), with group size varying slightly based on pace of recruitment. To optimize the pace of enrollment and trial completion, groups were comprised of immediate start and CG participants (who had already completed their waiting period).

### Training and Supervision

Prior to running the Bounce Back groups, the group facilitator and CRC were trained on the experiences of AYAs. CRC training included a general overview of common diagnoses, treatment trajectories, late effects, challenges, and stressors associated with the early posttreatment period. Additional instructional sessions included how to engage AYAs, manage distressed or frustrated AYAs, communicate with AYAs and parents of different cultural backgrounds, and communicate with providers about eligible AYAs.

The Bounce Back group facilitator was a doctoral-level clinical psychologist trained to deliver the SMART-3RP. This foundational training was supplemented by additional trial-specific training to review manual adaptations specific to Bounce Back, learn study protocols, and review physical and emotional challenges related to cancer treatment. Additional didactics included interpersonal skills to deliver a virtual group program and manage group dynamics over videoconferencing. The group facilitators were instructed to strictly adhere to the treatment protocol, which included reviewing previous material at the start of each session, covering all prescribed educational material, and leading in-session exercises.

Prior to the group start date, the study PI reviewed any potential participant concerns with the group facilitator to ensure proper implementation and tailoring of the program protocol. During the program, the study team (PI, group facilitator, and CRC) met weekly for clinical supervision and to review any changes or variations in program content delivery (ie, due to time constraints) and fidelity, as well as to troubleshoot barriers to participant engagement, attendance, and group cohesion.

### Fidelity

We developed a REDCap fidelity database to track Bounce Back program content and program engagement. The database included fields to track (1) session duration, (2) program content and exercises covered, (3) between-session practice goals assigned, (4) notable tech issues, (5) group cohesion, (6) participant attendance, and (7) participant engagement. Group cohesion was assessed through an investigator-developed measure asking the facilitator to rate the presence of the Group Therapeutic Factors (eg, altruism, interpersonal learning) defined by Yalom and Leszcz [35] on a 3-point Likert scale (not at all present, somewhat present, highly present). Immediately after each session concluded, the group facilitator tracked these items on the fidelity database, which was reviewed by the study PI to ensure protocol fidelity.

### Outcome Measures and Assessment Periods

Participants completed electronic study surveys via REDCap at 3 time points. Each self-report survey took approximately 15 minutes to 20 minutes to complete. The baseline (T0) survey was completed approximately 2 weeks before a new group was scheduled to begin. The time point 1 (T1) survey was completed up to approximately 12 weeks (±2 weeks) after T0, and time point 2 (T2) was completed up to approximately 24 weeks (±2 weeks) after T0. The CRC followed up weekly with participants who had outstanding surveys using multiple modalities. If the CRC could not reach a participant with incomplete surveys, the group facilitator also called and left a voicemail for participants to encourage survey completion. Outcome measures collected at screening and in study questionnaires are detailed in Table 3.

**Table 3:** Outcome measures.

Data			At screening		At baseline		At program		At postprogram		At 3-month follow-up
Date of birth			x		—^a^		—		—		—
Gender			x		—		—		—		—
Languages spoken			x		—		—		—		—
Cancer diagnosis			x		—		—		—		—
Date of diagnosis			x		—		—		—		—
Treatment type(s)			x		—		—		—		—
Date of treatment completion			x		—		—		—		—
Demographic factors			—		x		—		—		—
**Psychosocial measures**											
	Visual Analog Scales (VAS): Stress and Distress	—		x		—		x		x	
	Intolerance of Uncertainty Scale (IUS-12)	—		x		—		x		x	
	Measure of Current Status – Part A (MOCS-A)	—		x		—		x		x	
	Patient-Reported Outcomes Measurement Information System (PROMIS) Measures (PROMIS anxiety - short form 4a; PROMIS depression - short form 4a; PROMIS anger - short form 5a; PROMIS fatigue - short form 7b; PROMIS sleep disturbance - short form 8a; PROMIS social isolation - short form 4a)	—		x		—		x		x	
	Coping Self-Efficacy Scale (CSES)	—		x		—		x		x	
	Relaxation response (RR) practice	—		x		—		x		x	
	Health behavior questions	—		x		—		x		x	
	Penn State Worry Questionnaire (PSW-Q)	—		x		—		x		x	
	Interpersonal Reactivity Index (IRI)	—		x		—		x		x	
	Current Experiences Scale (CES)	—		x		—		x		x	
Hair cortisol measurement			—		x		—		x		—
Program acceptability questionnaire			—		—		—		x (PG^b^ only)		x (CG^c^ only)
COVID-19 supplementary questions			—		x		—		x		x
Optional weekly RR practice logs			—		—		x		—		—
Optional exit interview			—		—		—		—		x (after completion of all study measures)

^a^Not measured at this time point.

^b^PG: program group.

^c^CG: waitlist control group.

### Primary Outcome Measures

The primary aim of the study is to determine the feasibility and acceptability of the Bounce Back program.

#### Feasibility

Feasibility metrics were modeled after resiliency studies led with survivors and other medical populations [36,37]. We evaluated program feasibility by examining several process variables, including rates of study eligibility (percent of individuals who were eligible), recruitment (number of eligible individuals who expressed interest in our study), enrollment (percent of eligible pool who consented and enrolled), and retention (percent of enrollees who completed the follow-up). Our primary measure of feasibility was determined by the proportion of patients who completed the program, defined as participating in 6 out of 8 sessions. We documented reasons for ineligibility and refusal as well as sociodemographic characteristics, medical history, and cancer characteristics of refusers.

#### Acceptability

Program acceptability was assessed at the postprogram data collection period (T1 for PG, T2 for CG) with 5 questions on an acceptability questionnaire rated on a 4-point Likert scale (1=not at all to 4=very much). Items prompted participants to rate the extent to which they found the Bounce Back program to be (1) enjoyable, (2) helpful, (3) applicable or relevant (ie, is it appropriate and applicable), (4) convenient (ie, in regard to delivery modality), and (5) likelihood of future use (eg, “Will you continue to use RR strategies in the future?”). Treatment satisfaction was assessed by items on the acceptability questionnaire, which asks participants to rate their level of satisfaction with the following items using a 4-point Likert scale (1=not at all satisfied to 4=very satisfied): (1) treatment structure, (2) treatment timing (ie, early survivorship period) and (3) treatment content. We qualitatively explored overall satisfaction by asking 3 open-ended questions regarding treatment likes, dislikes, and recommendations. Additional acceptability data are collected in the optional qualitative exit interview.

### Secondary (Exploratory) Outcome Measures

#### Stress Coping: Measure of Current Status

The Measure of Current Status Part A (MOCS-A) is a 13-item measure that assesses participants’ self-reported ability to deal with daily stresses. Composite scores range from 0 to 52, with higher scores demonstrating greater self-perceived confidence in handling daily stressors. The MOCS-A has 4 subscales that can be analyzed: relaxation, awareness of tension, assertiveness, and coping confidence [38].

#### Resilience: Current Experiences Scale

Resilience was measured using 18 items from the Current Experience Scale (CES). The questionnaire reflects current self-perceived functioning in the domains of appreciation for life, adaptive perspectives, personal strength, spiritual connectedness, relating to others, and health behaviors. For each item, responses range from 0 (not at all) to 5 (a great deal). Composite scores range from 0 to 90, with higher scores indicating resiliency; greater scores on each of the 6 subscales indicate greater resiliency [39].

#### Stress, Distress: Visual Analogue Scale—Stress, Distress

The visual analogue scale (VAS)-Stress is a 1-item scale asking individuals to rate their current level of stress. The VAS-Distress is a 1-item scale asking individuals to rate their current level of distress on a scale of 0 to 10. Higher scores on each scale indicate greater levels of the construct being measured [40].

#### Patient-Reported Outcomes Measurement Information System Measures

Patient-Reported Outcomes Measurement Information (PROMIS) measures evaluate and monitor physical, mental, and social health in adults and children. The following subscales were utilized: PROMIS ED Anxiety – short form 4a, PROMIS ED depression – short form 4a, PROMIS ED anger – short form 5a, PROMIS fatigue - short form 7b, PROMIS sleep disturbance - short form 8a, PROMIS Social Isolation - short form 4a. PROMIS measures were scored by the HealthMeasures Scoring Service using response pattern scoring. PROMIS raw scores are converted into T-scores for each participant and compared to US population averages.

#### Worry: Penn State Worry Questionnaire

The Penn State Worry Questionnaire (PSWQ) is a 16-item measure used to assess worry. It is rated on a 5-point scale ranging from 1 (not at all typical of me) to 5 (very typical of me); select items are reverse scored. Total scores range from 16 to 80, with higher scores indicating greater worry [41].

#### Uncertainty Tolerance: Intolerance of Uncertainty Scale

The Intolerance of Uncertainty Scale (IUS-12) is a short version of the original 27-item Intolerance of Uncertainty Scale [42] that measures responses to uncertainty, ambiguous situations, and the future. The 12 items are rated on a 5-point Likert scale ranging from 1 (not at all characteristic of me) to 5 (entirely characteristic of me). The IUS-12 is scored on a scale from 12 to 60, with greater scores indicating greater intolerance of uncertainty. IUS prospective and inhibitory subscale scores will also be examined [43].

#### Perspective Taking: The Interpersonal Reactivity Index Perspective-Taking Subscale

The Interpersonal Reactivity Index (IRI) perspective-taking subscale is a 7-item measure that assesses the tendency of an individual to take on the perspective of another in daily life. Items are rated on a scale of 0 (does not describe me well) to 4 (describes me very well). We used 6 of the 7 items in the subscale. Total scores range from 0 to 24, with higher scores indicating greater perspective-taking ability [44].

#### Coping Self-Efficacy: Coping Self-Efficacy Scale

The Coping Self-Efficacy Scale (CSES) is a 26-item measure of self-perceived efficacy for coping with challenges and threats. Respondents are asked to rate their confidence performing adaptive coping behaviors (ie, talking positively to oneself) on a scale of 0 (Cannot do at all) to 10 (Certain can do). Scores range from 0 to 260, with higher scores indicating greater coping self-efficacy [45].

#### Relaxation Response Practice: RR Practice Measure

A single-item, investigator-developed measure was administered to assess frequency of self-guided RR exercise practice. Participants were asked to describe the frequency of their RR exercise (eg, mindfulness, guided imagery, deep breathing) practice on the following scale: Daily, A few times per week, Once or twice a month, or Never.

#### Health Behaviors: Health Behavior Questionnaire

The Health Behavior Questionnaire is an investigator-developed questionnaire designed to assess habits related to substance use, exercise, and nutrition.

#### Impact of COVID-19: COVID-19 Measure

This measure was added mid-study to account for any COVID-19–related stressors that occurred during study participation that may have influenced prior survey responses. Participants were asked to report on their COVID-19–related concerns, COVID-19–related lifestyle changes (ie, diet, sleep), changes in stress level, changes in cancer-related concerns, and more broadly how the virus was impacting their life.

#### Hair Cortisol Measurement

Participants were asked to provide hair samples to measure potential changes in cortisol (ie, a stress hormone). We found that hair cortisol sampling was feasible in a similar behavioral trial conducted with posttreatment lymphoma survivors [46]. Participants were instructed to provide 1 hair sample preprogram (T0 for PG, T1 for CG) and 1 sample at the end of the program (T1 for PG, T2 for CG). The CRC sent detailed hair sampling instructions and stamped, pre-addressed envelopes to facilitate returns. Participants were instructed to cut a small sample of hair (approximately 150 strands, about the diameter of a pencil eraser) from the back of their head, as close to the scalp as possible. They were asked to tie the strands near the scalp end, place the sample in aluminum foil, and mail back to the research team. The hair sampling instructions also included 6 questions about hair care, exercise, and glucocorticoid use, as these can affect hair cortisol measurements. Hair samples were not collected from participants who had taken glucocorticoid medications (eg, prednisone) within the past 3 months, as these medications can suppress endogenous cortisol levels or cause cortisol measurements to be inaccurate. We tracked the reasons why any hair samples were not collected to inform the feasibility and acceptability of hair cortisol collection and analysis for this population. We also collected feedback and perceptions of hair sampling measures at study completion. Prior to processing, samples remained wrapped in aluminum foil, labeled with a study ID, and stored at room temperature in a padded envelope. Hair samples were processed by Dr. Jerrold Meyer’s laboratory at the University of Massachusetts, Amherst using an ELISA assay kit.

#### Qualitative Exit Interviews

Qualitative data collection for this trial is ongoing. A randomly selected subset of 20 participants will be invited to participate in one-on-one exit interviews after study completion. To ensure inclusivity and take into account the effects of the COVID-19 pandemic on the program delivery, the sample will be stratified based on the following characteristics: (1) gender, (2) race (ie, non-Hispanic White; survivors of color), and (3) time of study participation (ie, before, during, or after onset of COVID-19 pandemic). Exit interviews may be completed via Zoom videoconferencing to explore additional barriers or facilitators to study participation, treatment adherence, program engagement, and study completion. Participants will be asked more detailed information about perceptions of the treatment and preferences for further adaptation after having participated in the program. A series of questions will be asked about using social media outreach for future research recruitment. We will also ask participants to report on how COVID-19 may have impacted their stress levels or ability to participate in the program. These interviews will be audio-recorded and qualitatively analyzed for themes that will help to determine whether treatment modifications are needed in future work. It is estimated that the interviews will take approximately 45 minutes to complete. Participants will be informed that the qualitative exit interviews are an optional portion of the study but if completed, will result in additional compensation (US $30).

### Safety

#### Data Safety Monitoring Plan

The PI monitored the safety of this trial and complied with reporting requirements. All adverse events were reported to the IRB within 24 hours. Study recruitment, enrollment, and retention were reviewed by the PI and CRC weekly. The PI’s mentor, co-mentors, consultants, and scientific advisors functioned as a Data Safety and Monitoring Board. This group convened on a semi-annual basis to monitor study participant safety and to review study progress and other study-related events (including, but not limited to, enrollment, recruitment, retention, and adverse events). During these meetings, any study-related concerns were reviewed, and as needed, an action plan was established. The outcome of these meetings and proposed action plans were summarized and distributed to all mentors, consultants, and scientific advisors. The PI and her team also met quarterly with collaborators within the CONNECCS network to review study progress, request referrals, and discuss other study-related activities and events. Study updates were summarized and distributed to all CONNECCS collaborators following the quarterly meetings.

#### Privacy and Confidentiality

We instructed participants to maintain the confidentiality of the group by not discussing anything that occurred in the group with anyone outside of the group. Group privacy and confidentiality were discussed at the first session and in the subsequent session to reinforce practice. Careful attention was taken during the informed consent process to explain the limits of confidentiality. Participants were advised to wear headphones and sit in a quiet place to protect their own and other group members’ privacy. All data and personal information created by this research study were stored in password-protected computer files accessible only to study staff and stored on a secure drive only accessible by members of the research team.

### Statistical Analysis

The primary study endpoints are the feasibility and acceptability of the Bounce Back program for AYA cancer survivors who are within 5 years posttreatment completion. Data analysis is ongoing, and the data analysis plan is reported in the following sections.

#### Sample Size and Power Calculations

Consistent with best practices in treatment development, the aim of this pilot is to establish the feasibility and acceptability of a stress management and resiliency program for early posttreatment AYAs [37,47]. We consider 75% session completion rate (approximately 6 of 8 sessions) as a threshold for program completion. As such, we consider 60% of participants reaching the threshold to establish program feasibility. With a sample size of 60 participants, we would have 80% power to demonstrate a difference of 15% from our preset criterion with a one-sided significance level of .05. Therefore, we believe our sample size of 72, accounting for 10%-15% attrition based on prior trials [46], will be sufficient to answer our questions about feasibility and acceptability.

#### Primary Analysis Plan

Descriptive statistics, including means, frequencies, and ranges, will be used to describe the sample and to summarize feasibility, acceptability, and program satisfaction. Feasibility outcomes will be assessed by determining the proportion of individuals who were recruited, screened, and enrolled in the study. Response frequencies will summarize reasons for ineligibility and refusal. We will also determine the proportion of enrolled participants who complete the program. Participants who complete at least 75% of the treatment sessions (6 of 8 sessions) will be identified as treatment completers. We will examine the proportion of individuals who attend each session. For acceptability, response frequencies will summarize quantitative feedback on the acceptability questionnaire. Together with qualitative feedback from the exit interviews, this information will be used to inform the feasibility and acceptability of the program.

#### Exploratory Analysis Plan: Psychosocial Measures

Preliminary outcome data may be used to inform future assessment instruments and methods. We may also conduct exploratory hypothesis testing to examine preliminary changes in our proposed program targets (changes in psychosocial outcomes, including mindfulness, depressed mood, anxiety, and stress). A priori statistical tests of program-related changes will be planned for a future efficacy trial of this program. First, we will examine the frequency distributions of all variables. Potential variables of interest (eg, gender, history of RR practice) will be included as covariates if they are significantly correlated with each outcome of interest at *P*<.25. We will also compare the baseline characteristics of completers versus study noncompleters. The primary analysis will be a completer analysis limited to those with complete data, and we will conduct a sensitivity analysis using multiple imputation for missing data. For our exploratory psychosocial outcomes, we will examine between-group differences in change scores from enrollment (T0) to T1 (posttreatment for PG, 3 months postenrollment/baseline #2 for CG). To further explore preliminary efficacy, we will evaluate within-group changes from pre- to postprogram (using T0 to T1 data for the PG and T1 to T2 data for the CG) for each condition separately and then for both groups combined. Finally, within the PG only, we will explore potential maintenance of program benefits with a repeated measures analysis of variance (ANOVA), including the 3 survey time points. Exit interviews will be audio-recorded and transcribed; NVIVO software will be utilized in the thematic analysis, which will be led by members of the study staff under the mentorship of the study PI. Coders will meet on a weekly basis to discuss the coding framework, categories, and coding plan. To ensure coding reliability, coding discrepancies will be resolved through discussion and comparison of raw data. Coding will continue until a high level of reliability (kappa ≥0.80) is established.

#### Exploratory Analysis Plan: Hair Samples

We will examine the feasibility, acceptability, and preliminary effects of collecting hair samples to examine changes in stress reactivity. Feasibility metrics for the hair sampling include hair return rates. For measures of acceptability, response frequencies will summarize quantitative feedback from the acceptability questionnaire about the acceptability of hair collection procedures. Hair cortisol samples will be analyzed in a laboratory, and group differences in hair cortisol concentration at T1 will be examined using independent samples *t* tests. Pearson correlation or Spearman rank correlation will examine the association of hair cortisol concentration with each of our psychological outcomes, controlling for potential confounders

#### Missing Data

We will assess whether the mechanism of missing data is missing at random. We will explore differences between study completers and noncompleters on participant demographic and other relevant variables to inform the next phase of this trial. We will perform sensitivity analysis using (1) a completer analysis limited to those who have complete data and (2) multiple imputations for missing data [48].

## Results

This project was part of a 5-year grant funded by the NCI in July 2017. The randomized controlled trial portion of the Bounce Back study occurred from July 2019 to March 2021. Of the 72 participants who enrolled in the study, 70 remained eligible (2 had a recurrence before groups began), and 64 initiated treatment. We ran 9 consecutive 8-week Bounce Back groups from July 2019 through December 2020. Quantitative data collection is complete, and qualitative exit interview data collection is ongoing but expected to be completed by June 2022. Data analysis is ongoing, and results are expected to be published in peer-reviewed journals and presented at local, national, or international meetings in the coming year(s).

## Discussion

This paper details the study protocol and methodology for a pilot randomized waitlist-control trial to examine the effects of a virtual program (Bounce Back) aimed at promoting stress management, resiliency, and coping among posttreatment AYAs.

Survivors of cancer diagnosed during adolescence and young adulthood are a largely understudied and underserved population. A cancer diagnosis during this life stage can cause significant disruption in several key life domains. Rates of stress and distress are high among this population, who often have poor health-related quality of life in the years after treatment [7-10]. Despite the prevalence of these challenges, few AYAs receive mental health services after treatment completion [2,49]. Without sufficient psychosocial supportive care, the rates of distress, morbidity, and mortality in this population will remain high. There are currently few evidence-based programs for AYAs in the years following treatment that tackle the key transitional issues they face [14,16,17,25,26]. As such, programs that promote stress management, coping, and connection among this population are warranted.

Other psychosocial programs targeting AYAs have been individually delivered, did not include mind-body skill acquisition, or focused on teaching a single skill (ie, mindfulness, positive psychology) for stress reduction [12-17,25,26]. Few have targeted a wide range of AYAs, particularly during the early posttreatment period. The use of both quantitative (surveys) and qualitative methods (exit interviews) will help us gain a richer understanding of AYAs’ experience in the program and its impact on their stress coping and psychological well-being. The wait-list control trial design allowed us provide support to all research participants who sought help while maintaining a nonprogram comparison group to enhance scientific rigor.

Historically, low research participation and a wide geographic distribution have made it difficult to identify AYAs and provide targeted treatment [32,33]. The virtual modality of the Bounce Back program promoted accessibility of our research study to participants who may not have been able to receive mental health care due to travel, financial, or health-related barriers. By using social media as a research recruitment tool and opening recruitment outside of our direct hospital system, we aimed to reach a more diverse and representative sample. With few restrictions in our inclusion and exclusion criteria, we ensured that the program was accessible to as many AYAs as possible. Notably, the Bounce Back trial and procedures spanned the timeframe of the COVID-19 pandemic. We did not cease operations of the trial during this time, acknowledging the need to support AYAs during a period of unprecedented uncertainty and health-related anxiety affecting individuals around the globe. To tease out the impact of the pandemic on our study outcome measures, we included a COVID-19 measure to our survey battery and exit interview for the subset of participants who were in the trial after the pandemic onset.

The Bounce Back program was adapted from an existing evidence-based mind-body program, the SMART-3RP [27]. The SMART-3RP has been proven to decrease stress and improve psychological and physical health symptoms among several different patient populations [46,50-53]. Offering a tailored mind-body program centered on the RR to AYAs may help mitigate the negative psychological and physiological effects of stress in the early posttreatment period. Additionally, few studies have examined hair cortisol as a biomarker of stress in the AYA population.

This study protocol does have some limitations. One limitation of this study is the potential for attrition. We expected that the rate of attrition would be similar to other randomized controlled trials of cognitive behavioral programs for children and adolescents with chronic illness [54].

Due to the waitlist-control trial design, participants had to wait up to 3 months before starting the treatment program. Some CG participants became unreachable during this waiting period prior to program participation. Additionally, with the AYA population, academic course schedules, work obligations, and extracurricular activities could conflict with the scheduled group program. Given that AYA schedules were often fluctuating, we enrolled individuals who stated their interest in participating regardless of their availability. This flexibility may have elevated our rate of attrition, resulting in some AYAs becoming unavailable for program scheduling after enrolling. Another limitation was exclusion of individuals who did not have access to appropriate technology, working internet, or an electronic device with a webcam to attend the virtual group sessions. However, in today’s digitally interconnected society, we did not anticipate that this requirement would preclude many AYAs from participating. Despite these limitations, our study participation rate remained quite high and was higher than those commonly noted in other behavioral trials [12,14]. Finally, we excluded non-English-speaking participants as the study measures and program were targeted towards an English-speaking audience. We hope to open future studies of the Bounce Back program to non-English speakers.

Our study results will add to the existing literature surrounding the feasibility and acceptability of delivering virtual programs to AYAs in the early posttreatment period. We will learn if the Bounce Back program can improve stress coping, distress, and psychological well-being in this understudied population. Our findings will help us gain a richer understanding of the psychosocial functioning of early AYAs as well as their perceptions surrounding mind-body and psychosocial supportive group programs. If the Bounce Back program is found to be efficacious, it will inform the design of future psychosocial programs for this population. If any psychosocial outcomes do not improve, it will allow us to determine what constructs to target in future programs for AYAs.

## Abbreviations

ANOVA: analysis of variance
AYAs: adolescents and young adults
CES: Current Experiences Scale
CG: waitlist control group
CONNECCS: Consortium for New England Childhood Cancer Survivors
CRC: clinical research coordinator
CSES: Coping Self-Efficacy Scale
DFCI: Dana-Farber Cancer Institute
EHR: electronic health records
IRB: Institutional Review Board
IRI: The Interpersonal Reactivity Index
IUS-12: Intolerance of Uncertainty Scale
MGH: Massachusetts General Hospital
MOCS-A: Measure of Current Status Part A
NCI: National Cancer Institute
PG: program group
PI: principal investigator
PROMIS: Patient-Reported Outcomes Measurement Information System
PSW: Penn State Worry Questionnaire
REDCap: Research Electronic Data Capture
RR: relaxation response
SMART-3RP: Stress Management and Relaxation Training - Relaxation Response Resiliency Program
VAS: visual analogue scale
